# NPBIP: predicting binding preferences of uncharacterized nucleic-acid-binding proteins

**DOI:** 10.1093/bioinformatics/btag232

**Published:** 2026-07-07

**Authors:** Noam Shimshoviz, Safwan Butto, Yaron Orenstein

**Affiliations:** The Mina and Everard Goodman Faculty of Life Sciences, Bar-Ilan University, Ramat Gan, 5290002, Israel; Department of Computer Science, Bar-Ilan University, Ramat Gan, 5290002, Israel; The Mina and Everard Goodman Faculty of Life Sciences, Bar-Ilan University, Ramat Gan, 5290002, Israel; Department of Computer Science, Bar-Ilan University, Ramat Gan, 5290002, Israel

## Abstract

**Motivation:**

Nucleic-acid-binding proteins (NBPs) are crucial regulators of gene expression, recognizing specific RNA or DNA binding sites. While high-throughput experiments have generated vast amounts of binding data, a significant challenge remains in predicting binding affinities of a novel query NBP to any nucleic-acid sequence. Current computational methods often require prior experimental data for the query NBP or are limited to predictions over predefined short RNA or DNA sequences.

**Results:**

We present New Protein Binding Intensity Predictor (NPBIP), a new method to predict the binding of a query NBP to any RNA or DNA sequence by integrating two complementary components: (i) a similarity-based method that computes a weighted mean of binding predictions over the training NBPs; and (ii) a deep-learning model that combines a large protein language model with a hybrid convolutional-transformer network to predict binding directly. We trained and evaluated NPBIP on 420 RNA-binding protein (RBP) experiments and 464 DNA-binding protein (DBP) experiments. NPBIP significantly outperformed each of its components and all competing baselines, achieving a mean Pearson correlation of 0.414±0.20 and 0.581±0.22 over the RNA- and DNA-binding experiments, respectively. This prediction performance was statistically comparable to an experimental *k*-mer upper bound over RBPs and statistically superior to an upper bound over DBPs. Furthermore, our interpretability analysis demonstrates that NPBIP recovers canonical binding motifs for both RBPs and DBPs, providing biological validation for the model’s predictions.

**Availability and implementation:**

Source code and datasets are publicly available at https://github.com/OrensteinLab/NPBIP.

## 1 Introduction

Nucleic-acid-binding proteins (NBPs) are primary regulators of gene expression. Transcription factors (TFs), a class of DNA-binding proteins (DBPs), modulate transcription by binding specific DNA sequences near target genes to influence RNA polymerase recruitment (Chen and Rajewsky 2007). RNA-binding proteins (RBPs) govern post-transcriptional regulation, including splicing, stability, and translation (Glisovic *et al.* 2008). This precise control depends on NBPs recognizing specific binding sites (BSs) according to distinct preferences.

Due to the critical roles of NBPs, a variety of high-throughput experimental assays have been developed to characterize their binding preferences. *In vivo* assays, such as ChIP-seq, CUT&Tag, and eCLIP, measure binding within the native cellular context but are influenced by factors like chromatin accessibility, co-binders, and cell-type-specific conditions, making it difficult to disentangle the intrinsic binding preferences of NBPs from the cellular context (Ben-Bassat *et al.* 2018). In contrast, *in vitro* assays provide a controlled environment to infer intrinsic binding preferences independent of the cellular context. Microarray-based methods, such as RNAcompete (Ray *et al.* 2013) and protein-binding microarrays (PBMs) (Berger *et al.* 2006), measure binding over large RNA and DNA probe libraries, respectively, and have been applied to hundreds of NBPs.

The experimental datasets produced by these high-throughput *in vitro* assays have enabled the development of computational models to represent protein–nucleic-acid binding preferences, often summarized as a BS motif. The most widely used model is the position weight matrix (PWM), which represents a motif as a 4×k matrix of nucleotide frequencies at each position. PWMs are interpretable and easily visualized as sequence logos, but their assumption of position independence often oversimplifies real binding preferences (Carlson *et al.* 2010). A more expressive alternative is the complete *k*-mer model, which assigns scores to every possible sequence of length *k*, capturing dependencies between positions. In recent years, deep-learning models have emerged as the state of the art in modeling RNA- and DNA-binding preferences (Horlacher *et al.* 2023). Deep-learning models can directly predict binding affinities over long sequences, offering greater flexibility and higher predictive performance compared to PWMs and *k*-mer models.

There are multiple tasks in predicting NBP binding to nucleic-acid sequences. The basic *NewSeq* task is to use a NBP’s experimental data to predict its binding to novel nucleic-acid sequences. State-of-the-art models for the *NewSeq* task, such as MultiRBP, use multi-task learning to leverage similarities across RBPs (Karin *et al.* 2021). The more challenging *NewPro* task is to predict the binding intensities of an NBP for which no experimental data is available. Approaches addressing this problem, such as Affinity Regression (Pelossof *et al.* 2015) and ProbeRating (Yang *et al.* 2020), can predict binding of uncharacterized NBPs but are limited to scoring only those nucleic-acid sequences present in the training datasets. Other methods, including JPLE (Sasse *et al.* 2025) and similarity regression (Lambert *et al.* 2019), predict the binding affinity of a new NBP to a predefined set of short sequences (e.g. all RNA 7-mers or DNA 8-mers). These methods rely on identifying similarities between the query NBP and the NBPs in the training set to make predictions. The most challenging and general *NewProNewSeq* task is to predict binding for a novel query NBP to any nucleic-acid sequence. Unfortunately, no current method is designed to generalize to this task in a principled and direct manner. While the complete *k*-mer model can be adapted to score longer sequences, it was not designed for this purpose, and its effectiveness remains untested.

In this work, we developed New Protein Binding Intensity Predictor (NPBIP) to solve the task of predicting binding of a novel query NBP to a given nucleic-acid sequence by integrating two components. The first component, SimBind, predicts the binding affinity of a query NBP by calculating its similarity to all training NBPs, and using these similarities to compute a weighted mean of the binding predictions made by each training NBP for the query nucleic-acid sequence. The second component, NucProNet, is a deep-learning model that utilizes a protein large language model, convolutional layers, and a transformer to directly predict the binding affinity for any NBP–nucleic-acid pair. Through comprehensive evaluation on both RBP and DBP datasets, we demonstrate that NPBIP significantly outperforms both its components and all *k*-mer-based methods. Moreover, we show that NPBIP’s performance is statistically comparable and superior to an experimental *k*-mer upper bound for RBPs and DBPs, respectively, indicating that NPBIP captures binding information lost by *k*-mer-based methods. Finally, our interpretability analysis demonstrates that NPBIP recovers canonical binding motifs of both RBPs and DBPs.

## 2 Materials and methods

### 2.1 Datasets

*RBP.* We downloaded the normalized RNAcompete data from two sources: 244 experiments from the original compendium of RBPs (Ray *et al.* 2013) and 176 additional experiments from the recent JPLE study (Sasse *et al.* 2025), together totaling 420 experiments over 380 RBPs (Table 1). Each experimental dataset contains binding measurements of a specific RBP over 241 357 30–41 nt-long RNA probes designed to cover together each RNA 9-mer at least 16 times (Ray *et al.* 2013). As in prior studies (Karin *et al.* 2021), we clipped extreme values by capping binding intensities at the 99.5th percentile within each experiment to reduce the effect of outliers and imputed missing values (0.80% of the measurements) using the mean binding intensity of each respective experiment (we expect negligible difference with alternative imputation strategies due to the very small fraction of missing data). For each experiment, we also downloaded its associated 7-mer binding model for our benchmarking of *k*-mer-based methods.

**Table 1:** Summary of dataset sizes used in this study.

Type	Database	No. of Exp.	No. of NBP	No. of Seq.
RNA (RNAcompete)	Compendium & JPLE	420	380	241 357
DNA (PBM)	UniPROBE	464	464	41 728

*DBP.* We downloaded the normalized PBM data from the UniPROBE database (Hume *et al.* 2015). Each experimental dataset contains binding measurements of a specific DBP over 41 728 36 nt-long DNA probes designed to cover together all DNA 10-mers. Since PBM experiments differ in the set of DNA probes they use, to ensure compatibility, we downloaded all experiments that used the most common probe set (termed “our all 10-mer de Bruijn sequences” in the UniPROBE database) and had the DBP sequence, the binding intensities, and an 8-mer binding model available. This set included 464 PBM experiments over 464 DBPs (Table 1). We imputed missing binding intensities (0.65% of the measurements) using the mean intensity of each respective experiment, and applied Z-score normalization per experiment, which is a common practice in PBM analysis to standardize binding intensities (Wang 2014). For each DBP, we also downloaded its TF family annotation from the UniPROBE database and its 8-mer binding model for our benchmarking of *k*-mer-based methods.

### 2.2 Training and evaluation

**Figure 1:**
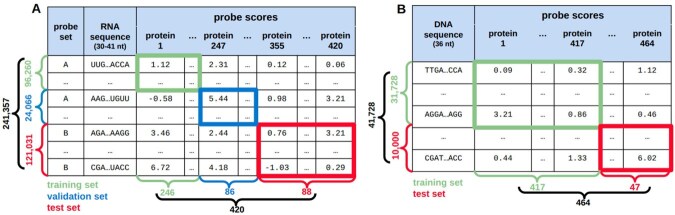
Overview of data partitioning for training, validation, and test sets. (A) RNAcompete data partitioning. RBPs and RNA sequences are partitioned into non-overlapping training, validation, and test sets. (B) PBM data partitioning. DBPs are partitioned using 10-fold cross-validation, while DNA sequences are divided into non-overlapping training and test sets.

To accurately simulate the *NewProNewSeq* task, we evaluated prediction performance by ensuring that both the test NBPs and the test nucleic-acid sequences are held out from model training and hyper-parameter optimization. For the RNAcompete dataset, we arbitrarily partitioned the 420 RBP experiments into training (246 experiments, 59%), validation (86 experiments, 20%), and test (88 experiments, 21%) sets, ensuring that all experiments for a given RBP are grouped together in the same set. The RNA sequences were originally partitioned by the developers of RNAcompete into two sets: Set A containing 120 326 sequences and Set B containing 121 031 sequences, with both sets having similar coverage of RNA 9-mers. We leveraged this partition, using 80% of Set A for training (96 260), 20% of Set A for validation (24 066), and the whole of Set B for testing (121 031) (Fig. 1A).

For the PBM dataset, since we did not perform a hyper-parameter search, we did not define a validation set. Instead, we gauged prediction performance by 10-fold cross-validation. In each cross-validation iteration, one fold (46−47 DBPs) served as the test set, while the remaining nine folds (417−418 DBPs) were used for training. For the DNA sequences, we used the first 31 728 probes of the PBM dataset for training and the last 10 000 probes for testing (Fig. 1B).

We selected hyper-parameters values based on the RNAcompete training and validation sets, and then applied them to both the RNA and DNA models without further tuning. This setup evaluates NPBIP’s ability to generalize across different experimental assays and nucleic-acid types without further hyper-parameter tuning. We evaluated model performance by Pearson correlation between predicted and observed binding intensities for each test NBP on the nucleic-acid sequence test set.

### 2.3 SimBind: the similarity-based component

**Figure 2:**
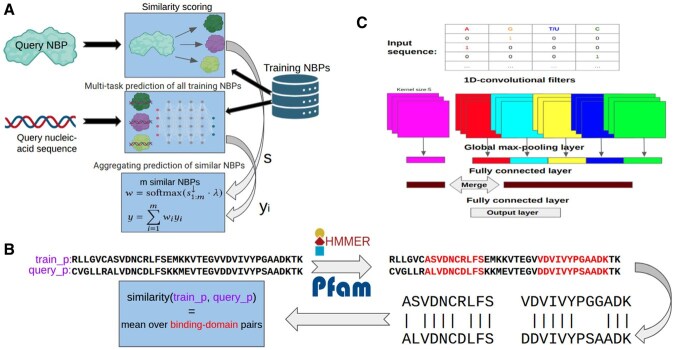
SimBind pipeline. (A) SimBind workflow: similarity scoring compares a query NBP to the training NBPs. Multi-task predictions are made for all training NBPs on the query nucleic-acid sequence. The predictions are then aggregated using a similarity-weighted sum. (B) Protein sequence similarity workflow: a pair of sequences (train_p, query_p) are processed via HMMER/Pfam to identify NBDs highlighted in red. The identified NBDs are pairwise aligned to compute NBDs similarity scores. These individual scores are then aggregated into a final protein similarity score. (C) Simplified top-view of MultiRBP and analogous MultiDBP architectures for multi-task binding predictions. The query nucleic-acid sequence is passed through convolutional, pooling, and fully connected layers, resulting in predictions for each training NBP.

SimBind predicts binding of a query RBP or DBP to a query RNA or DNA sequence, respectively. SimBind first calculates the similarity between the query NBP and the training NBPs. Then, SimBind predicts the binding intensity of each training NBP to the query RNA or DNA sequence (using a multi-task solution to the *NewSeq* task). Finally, SimBind outputs a weighted mean over the predicted binding intensities according to the calculated similarities (Fig. 2A).

#### 2.3.1 Computing protein sequence-similarity scores

SimBind follows the sequence similarity calculation of JPLE (Sasse *et al.* 2025) (Fig. 2B). SimBind first identifies all nucleic-acid-binding domains (NBDs) within the query NBP sequence using HMMER (Eddy 2009) over profile hidden Markov models from the Pfam database (Mistry *et al.* 2021). SimBind uses 44 and 20 Pfam models representing well-characterized DBPs and RBPs, respectively. For each identified binding domain (BD), SimBind extends the domain by 15 flanking amino acids on both sides to preserve the local context.

To compute the similarity between two NBPs, SimBind aligns each flanked BD in the query NBP to each BD in a training NBP using Needleman–Wunsch global alignment with the BLOSUM62 substitution matrix (gap opening penalty: −11; gap extension penalty: −1). For each BD-to-BD alignment, SimBind computes the similarity score as the fraction of exactly matched aligned residues over the total alignment length.

If both NBPs have the same number of BDs, the overall similarity score is computed as the mean over BD pairs matched by order. If the NBPs have a different number of BDs, SimBind computes the mean similarity score for all possible one-to-one order-conserving alignments of the smaller set of BDs to the larger set. SimBind selects the maximum score over these alignments as the similarity measure between the two NBPs.

#### 2.3.2 Binding prediction using a multi-task neural network

SimBind predicts the binding intensities of the training NBPs over the query nucleic-acid sequence using a multi-task neural network (Fig. 2C), which is considered the state of the art for the *NewSeq* task.

For RNA sequences, we used MultiRBP (Karin *et al.* 2021). We trained the model in two settings: (i) on the training set only as part of the hyper-parameter search. (ii) on the combined training and validation sets as part of the evaluation on the test set. For DNA sequences, we implemented MultiDBP, an analogue to MultiRBP, trained on the PBM data. We trained this model according to the 10-fold cross-validation: in each fold, we trained on the nine training folds to predict on the held-out fold. Both MultiRBP and MultiDBP receive a nucleic-acid sequence as input and output a vector of predicted binding intensities, one for each NBP it was trained on.

#### 2.3.3 Aggregating predictions of similar proteins

SimBind predicts the binding of the query NBP to a query nucleic-acid sequence by aggregating the predictions of similar NBPs. SimBind ranks the similarity scores *s* over all the training NBPs, and transforms the scores of the *m* most similar training NBPs $s_{1:m}^{↓}$ into a vector of coefficients via a λ-parameterized softmax function, $w=\text{softmax}(s_{1:m}^{↓}\cdot \lambda )$, where *m* and λ are hyper-parameters. SimBind then computes the final prediction *y* by a weighted sum over the individual predictions $y_{i}$ for NBP *i*: $y=\sum_{i=1}^{m}w_{i}y_{i}$.

### 2.4 NucProNet: the deep-learning component

**Figure 3:**
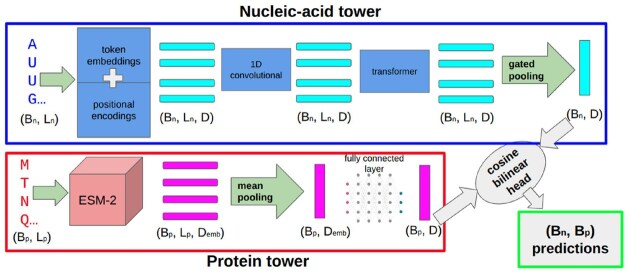
NucProNet overview. The nucleic-acid tower embeds the nucleic-acid sequence using token and positional encodings, processes it with 1D-convolutional layers and a transformer block, and uses gated pooling to produce a sequence embedding. The protein tower generates per-residue embeddings using ESM-2, which are then mean-pooled and passed through a fully connected layer to produce a protein embedding. A cosine bilinear head integrates the two embeddings to compute the final binding predictions. (Shape variables: $B_{n}$/$B_{p}$ = nucleic-acid/protein batch size, $L_{n}$/$L_{p}$ = sequence length, $D_{\mathrm{emb}}$ = ESM embedding dimension, *D* = final model dimension.).

NucProNet is a two-tower deep-learning model that receives a protein sequence and a nucleic-acid sequence as input, and outputs a single predicted binding affinity (Fig. 3).

The **protein tower** encodes the amino-acid sequence of an NBP using embeddings from the ESM-2 protein language model (Lin *et al.* 2023). These contextualized embeddings capture evolutionary, structural, and biochemical information learned from large-scale protein corpora. The per-residue embeddings are mean-pooled to obtain a sequence-level representation and then projected through a multilayer perceptron to produce the final fixed-length NBP embedding.

The **nucleic-acid tower** first embeds the nucleic-acid sequence using token embeddings for {A, C, G, U/T} and a learned positional encoding. This representation is processed sequentially by a hybrid architecture designed to capture features at multiple scales. First, three dilated Conv1D blocks are applied to effectively detect local sequence motifs. This is followed by a transformer encoder, which models complex, long-range dependencies between distant nucleotides within the sequence. Finally, a gated pooling layer aggregates the features into a sequence-level representation, which is then layer-normalized to produce the final RNA representation.

The resulting protein and nucleic-acid representations are integrated by a gated, low-rank cosine bilinear head that computes pairwise affinities between all proteins and nucleic-acid sequences in the batch. Full architectural details are available in Supplementary Methods, available as supplementary data at *Bioinformatics* online.

We trained NucProNet using the AdamW optimizer. The objective function was a weighted combination of Huber loss and a Pearson correlation-based loss:


$$L=\lambda_{1}{\text{Huber}}_{\delta =1.0}(\hat{y},y)+\lambda_{2}(1-r(\hat{y},y))$$

where $r(\hat{y},y)$ denotes the Pearson correlation coefficient between the vectors of predicted and observed binding intensities for all NBP–nucleic-acid pairs within a training batch. We selected this loss function was selected to directly optimize for Pearson correlation, our primary evaluation metric, while using the robust Huber loss for the regression component.

### 2.5 NPBIP: New protein binding intensity predictor

NPBIP integrates the predictions from SimBind and NucProNet. Since the two models produce scores on different scales, NPBIP first normalizes their outputs by converting each model’s vector of predictions for a given query NBP to Z-scores. The final NPBIP prediction for an NBP–nucleic-acid pair is the sum of the two Z-scores. Implementation details, including computing infrastructure and resource requirements, are in the Supplementary Methods, available as supplementary data at *Bioinformatics* online.

### 2.6 Benchmarking against competing methods

Since no current method is designed to solve the *NewProNewSeq* task directly, we benchmarked our models against methods predicting *k*-mer binding models. We compared NPBIP against three such methods. First, we evaluated our RBP models against JPLE, a state-of-the-art method for predicting 7-mer models for novel RBPs (Sasse *et al.* 2025).

Second, we created a SimBind-*k*-mer that adapts our SimBind method to predict a *k*-mer model. For a query NBP, it first identifies the *m* most similar training NBPs. To predict the score for a specific *k*-mer, SimBind-*k*-mer retrieves the score for that *k*-mer from the pre-computed, experimentally derived *k*-mer models of each of the *m* most-similar training NBPs. It then outputs a weighted mean of these *m* scores as the final predicted score for that *k*-mer. This process is repeated for all *k*-mers to generate a full predicted *k*-mer model.

Third, we created NucProNet-*k*-mer, a deep-learning model that shares the same two-tower architecture as NucProNet. We trained NucProNet-*k*-mer to predict the score for (NBP, *k*-mer) pair using the experimentally derived *k*-mer models as ground truth. To generate a complete *k*-mer model for a held-out test NBP, we used the trained model to predict the affinity for that NBP against every possible *k*-mer.

As an experimental upper bound, we used the experimentally derived *k*-mer model of the test NBP itself. This represents the best possible performance a method predicting a *k*-mer model could theoretically achieve, as it relies on having the ground-truth *k*-mer preferences for the test NBP.

To convert the *k*-mer model outputs for the *NewProNewSeq* task, we scored each probe by applying a sliding window of length *k* (7 for RNA, 8 for DNA). The final binding prediction for a probe is the mean over the scores of the *k*-mers contained within that sequence. A comparison of *k*-mer score aggregation strategies: mean, sum, max, and Boltzmann-sum, is described in the Supplementary Methods, available as supplementary data at *Bioinformatics* online, and its results are reported in Fig. 1, available as supplementary data at *Bioinformatics* online.

### 2.7 Interpretability analysis of NPBIP

To identify the key binding motifs driving NPBIP predictions, we used an occlusion analysis on NucProNet (Zeiler and Fergus 2014). We systematically masked short windows of the input nucleic-acid sequence and measured the resulting change in the predicted binding affinity. We selected a window size of k=3 nucleotides, consistent with methodologies in previous studies (Dutta *et al.* 2019). To generate a global consensus motif from these local attribution scores, we used F-MoDA (Yaish and Orenstein 2025). We extracted the top 100 highest-predicted sequences for a given NBP and computed their position-specific attribution scores. We then provided these attribution profiles as input to F-MoDA to generate the final consensus sequence logo. We used MoSBAT (Lambert *et al.* 2016) to quantify similarity between the recovered and ground-truth motif. We did not interrogate SimBind in the interpretability analysis, as it does not learn important features, but is rather based on similarity to training NBPs.

## 3 Results

### 3.1 NPBIP hyper-parameter optimization

SimBind and SimBind-*k*-mer have two hyper-parameters: the number of top similar NBPs (*m*) and the softmax scaling factor (λ). To determine their optimal values, we performed two separate systematic grid searches on the RNAcompete validation set. For SimBind, we tested 10 values for *m* (range 1–120) and 10 for λ (range 1–50), and evaluated the performance of each of the 100 (m,λ) combinations by mean Pearson correlation between predicted and observed binding intensities over all NBPs in the validation set (Fig. 2A, available as supplementary data at *Bioinformatics* online). This grid search identified m=14 and λ=8 as the optimal hyper-parameters. For SimBind-*k*-mer, the grid search identified m=14 and λ=15 as its optimal values (Fig. 2B, available as supplementary data at *Bioinformatics* online). We conclude that aggregating predictions from multiple NBPs yields improved performance compared to relying on the single most similar NBP (i.e. m=1). We fixed these respective optimal values for SimBind and SimBind-*k*-mer in all subsequent experiments, including training and testing on the PBM dataset (which was not part of this hyper-parameter search).

NucProNet and NucProNet-*k*-mer models have a large number of hyper-parameters, including learning rate, weight decay, embedding dimension, dropout rate, total number of training steps, and many more. We optimized these hyper-parameters for each model separately using the Optuna framework (Akiba *et al.* 2019). We performed the search for NucProNet on the RNAcompete dataset. In each Optuna trial, a model was trained with a specific set of hyper-parameters values on the training set. The model’s performance was then evaluated on the validation set, using the mean Pearson correlation as the objective metric for Optuna to maximize (Fig. 2C, available as supplementary data at *Bioinformatics* online). This search yielded an optimized set of hyper-parameters values (Table 1, available as supplementary data at *Bioinformatics* online). We performed a similar search for the NucProNet-*k*-mer model with the difference that the objective metric was the mean Pearson correlation over the 7-mer scores (Fig. 2D, available as supplementary data at *Bioinformatics* online). This search yielded an optimized set of hyper-parameters values (Table 1, available as supplementary data at *Bioinformatics* online). We set these respective values for NucProNet and NucProNet-*k*-mer in all subsequent experiments, including training and testing on the PBM dataset (which was not part of this hyper-parameter search).

Finally, we evaluated the strategy for integrating SimBind and NucProNet into the final NPBIP model. We compared a simple Z-score summation to a learned linear combination on our validation set. The optimized weights were nearly balanced ($w_{\mathrm{SimBind}}\approx 0.59$, $w_{\mathrm{NucProNet}}\approx 0.63$) and yielded a negligible performance gain of <0.001 in mean Pearson correlation. Thus, we chose a non-parametrized combination to avoid over-fitting.

### 3.2 NPBIP outperforms existing methods in RNA-binding prediction of novel RBPs

To evaluate performance in predicting binding intensities for query RBPs on unseen RNA sequences, we used the RNAcompete test set, which we defined to consist of 88 held-out RBPs and the 121 031 probes of Set B. We compared our three direct-prediction methods (SimBind, NucProNet, NPBIP) against three *k*-mer-based methods (JPLE, SimBind-*k*-mer, NucProNet-*k*-mer), and an experimental *k*-mer upper bound.

**Figure 4:**
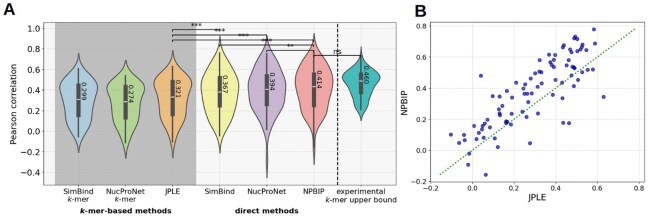
Performance in predicting RNA binding. (A) Distribution of Pearson correlations over the test RBPs. Mean correlations are written. (*** denotes *P*<.001, ** denotes *P<0.01, ns* = non-significant, Wilcoxon signed-rank test). (B) Pairwise comparison between NPBIP and JPLE.

NPBIP significantly outperformed all competing methods (Fig. 4A). NPBIP achieved a mean Pearson correlation of 0.414±0.20 which is significantly higher than its individual components, SimBind with 0.367±0.19 and NucProNet with 0.394±0.20 (*P*-value ≤ 4.41×10^−3^, Wilcoxon signed-rank test). This demonstrates a clear benefit from integrating the similarity-based and deep-learning components.

Our results show that direct prediction is more effective than *k*-mer-based methods. JPLE, NucProNet-*k*-mer, and SimBind-*k*-mer achieved a mean of 0.321±0.18, 0.274±0.17, and 0.299±0.17, respectively, which are all significantly lower than the performance of our three direct-prediction methods (*P*-value ≤ 9.95×10^−4^). When comparing the number of RBPs for which improved performance was gained, NPBIP outperformed JPLE in 69 out of 88 of the tested RNAcompete experiments (Fig. 4B). Remarkably, the performance of NPBIP closely approached, and was not statistically different from, the experimental *k*-mer upper bound which achieved a mean of 0.46±0.12 (*P*-value=.116). This upper bound represents an idealized scenario that uses the test RBPs’ own experimentally derived *k*-mer model, indicating that NPBIP’s predictive performance is comparable to a model with access to ground-truth information.

#### 3.2.1 Evaluating prediction of complete 7-mer RNA-binding models

For a direct comparison with JPLE, which was designed to predict a complete 7-mer model, we evaluated our methods on *k*-mer model prediction (Fig. 3, available as supplementary data at *Bioinformatics* online). Among the individual methods, JPLE achieved the highest mean Pearson correlation (0.605±0.35), followed by SimBind-*k*-mer (0.601±0.32), and NucProNet-*k*-mer (0.583±0.31) (*P*-value<7.28×10^−3^, Wilcoxon signed-rank test). Since an ensemble method performed better in *NewProNewSeq*, we evaluated ensembles by normalizing each individual method’s prediction vector to Z-scores and computing the sum of the normalized vectors. An ensemble of SimBind-*k*-mer and NucProNet-*k*-mer, achieved a mean Pearson correlation of 0.623±0.31, surpassing JPLE on mean though not significantly (*P*-value=.602). We further evaluated an ensemble method integrating JPLE, SimBind-*k*-mer, and NucProNet-*k*-mer. This ensemble achieved the highest performance with a mean of 0.633±0.31, significantly outperforming all three individual methods (*P*-value<5.95×10^−3^). Thus, in addition to NPBIP, we also achieved superior performance in predicting a *k*-mer model compared to the state of the art for this task.

### 3.3 NPBIP outperforms existing methods in DNA-binding prediction of novel DBPs

To evaluate performance in predicting binding intensities for query DBPs on unseen DNA sequences, we used the PBM dataset in a 10-fold DBP-level cross-validation. In each fold, the test set consisted of 42−43 held-out DBPs and the final 10 000 DNA probes. We compared our three direct-prediction methods (SimBind, NucProNet, NPBIP) against two *k*-mer-based methods (SimBind-*k*-mer, NucProNet-*k*-mer), and an experimental *k*-mer upper bound.

We first evaluated the performance of MultiDBP, a key component of SimBind, which had not been evaluated before [as opposed to MultiRBP which was shown to achieve state-of-the-art performance (Karin *et al.* 2021)]. We trained MultiDBP on the first 31 728 PBM probes and evaluated it on predictions for the final 10 000 test probes for the same DBPs. MultiDBP achieved a mean Pearson correlation of 0.727±0.16 over 464 DBPs (Fig. 4A, available as supplementary data at *Bioinformatics* online). More than 188 DBPs achieved a Pearson correlation >0.8, demonstrating the ability of MultiDBP to predict DNA binding over new DNA sequences.

When evaluating NPBIP prediction performance over DBPs, we found that NPBIP not only significantly outperformed all other methods but also achieved performance superior to the *k*-mer upper bound (Fig. 4B, available as supplementary data at *Bioinformatics* online). NPBIP achieved a mean Pearson correlation of 0.581±0.22, which is significantly higher than the 0.574±0.15 achieved by the *k*-mer upper bound (*P*-value=1.41×10^−2^, Wilcoxon signed-rank test). NPBIP achieved statistically superior performance compared to its individual components, SimBind with 0.556±0.22 and NucProNet with 0.544±0.23 (*P*-value≤7.21×10^−23^). Furthermore, NucProNet-*k*-mer and SimBind-*k*-mer achieved a mean of 0.404±0.19 and 0.449±0.20, respectively, which are all significantly lower than the performance of all three of our direct-prediction methods (*P*-value≤6.70×10^−33^).

**Figure 5:**
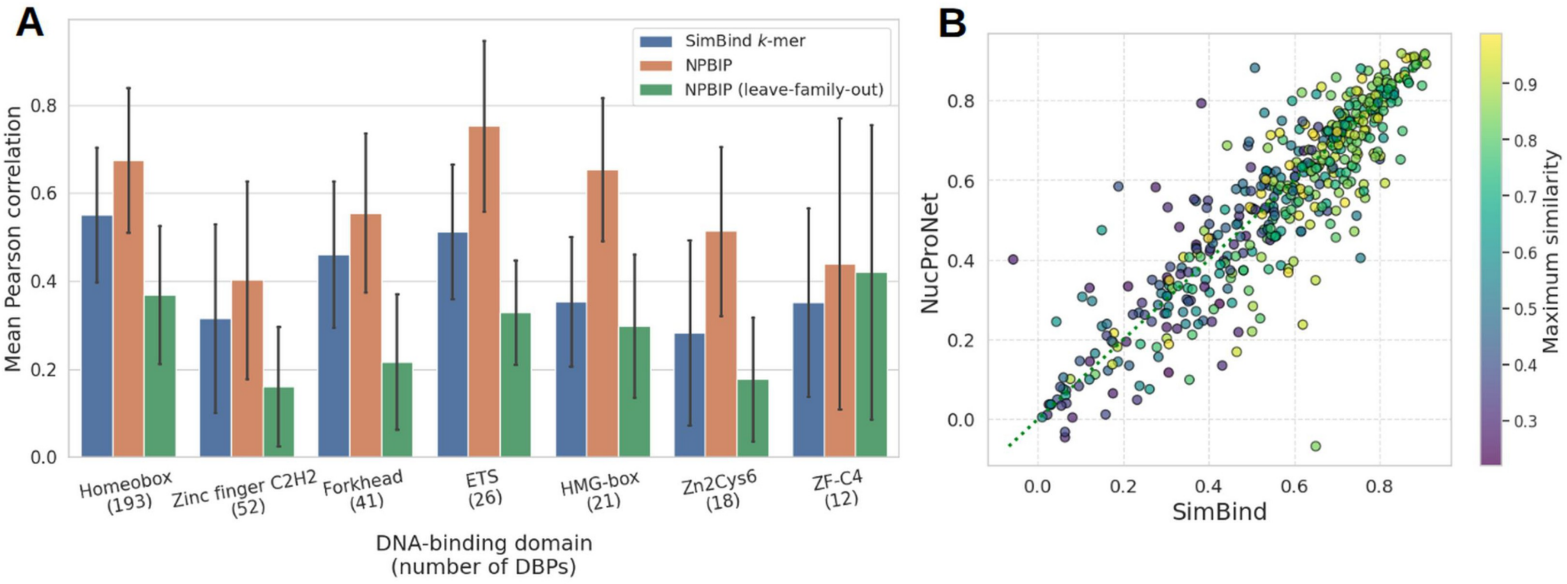
Prediction performance as a function of TF family and query NBP maximum similarity to the training NBPs. (A) Prediction performance over the seven TF families in the DBP dataset. Performance evaluated by mean Pearson correlation over the DBPs in the family with their total number in parentheses. (B) Per-query-NBP performance, as measured by Pearson correlation, of NucProNet versus SimBind, colored by maximum sequence similarity.

**Figure 6:**
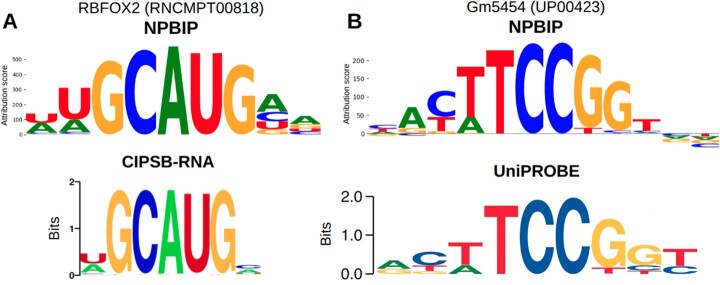
Interpretability analysis. (A) The aggregated binding motif for RBFOX2 recovered by NPBIP aligns with the known reference from CISBP-RNA. (B) The aggregated binding motif for Gm5454 recovered by NPBIP aligns with the known reference from UniPROBE.

We further evaluated all methods using Spearman correlation, Precision@100, and the Area Under the Precision-Recall Curve (AUPRC) for the top 1% of sequences. NPBIP outperformed all methods across all examined metrics for both RNA and DNA datasets (Figs 5–10, available as supplementary data at *Bioinformatics* online). In top-N retrieval tasks, NPBIP’s performance surpassed the experimental *k*-mer upper bound. Specifically, NPBIP achieved a mean Precision@100 of 0.424±0.21 for RNA and 0.364±0.22 for DNA, which are significantly higher than the respective *k*-mer upper bounds of 0.299±0.11 and 0.317±0.12 (*P*-value≤6.8×10^−4^, Wilcoxon signed-rank test).

### 3.4 NPBIP prediction performance by TF family

To test NPBIP’s prediction performance across TF families, we analyzed its performance within seven major TF families present in the PBM dataset with NPBIP and SimBind-*k*-mer, the best performers among direct-binding and *k*-mer-based methods, respectively (Fig. 5A). We evaluated performance by the mean Pearson correlation achieved over all DBPs within a family when they were held out as part of the test set in our 10-fold cross-validation. We also performed a leave-family-out analysis, in which we retrained NPBIP while excluding the target family from the training set.

NPBIP consistently outperformed SimBind-*k*-mer across all examined TF families. NPBIP achieved high prediction performance in the homeobox and ETS families, achieving a mean Pearson correlation of 0.675±0.17 and 0.757±0.19, respectively. The high performance in the Homeobox family may be explained by the fact that it is the most popular TF family in the dataset with 193 DBPs out of 464, which increases the likelihood of identifying highly similar training DBPs to the query DBP. Conversely, the ETS family, comprising only 26 DBPs, also yielded high performance, which may be attributed to the high degree of sequence conservation within its DNA-BD, leading to good generalization even when fewer training DBPs are available (Hollenhorst *et al.* 2011). As expected, in a leave-family-out setting, performance decreased across all families, highlighting the impact of family-level information (Fig. 5A).

### 3.5 Impact of protein similarity on prediction performance

We next tested how prediction performance is affected by a query NBP’s similarity to the training set. To do this, we calculated two values for each test NBP: (i) the Pearson correlation achieved for it, and (ii) its maximum similarity score to any NBP in the training set. We observed a Pearson correlation of 0.63 (*P*-value=3.9×10^−11^) for RBPs (Fig. 11A, available as supplementary data at *Bioinformatics* online) and 0.50 (*P*-value=2.3×10^−30^) for DBPs (Fig. 11B, available as supplementary data at *Bioinformatics* online). This trend is expected for SimBind, which explicitly uses similarity. However, we found this correlation also holds for NucProNet, which does not use explicit similarity information. As illustrated for the DBP dataset (Fig. 5B), DBPs with high similarity show high performance for both SimBind and NucProNet. Conversely, DBPs with low similarity perform poorly with both. This demonstrates that NucProNet also implicitly learns to generalize more effectively for NBPs that are similar to those in the training set, reinforcing that similarity is a key driver of performance for both of NPBIP’s components.

### 3.6 NPBIP learns intrinsic binding preferences

To verify that NPBIP learned biologically relevant patterns, we applied an interpretability analysis to the NucProNet component. We first analyzed the *Homo sapiens* RBP RBFOX2 (RNCMPT00818). We computed attribution scores of the 100 top-predicted testing sequences and observed distinct peaks of high importance (Fig. 12A, available as supplementary data at *Bioinformatics* online). We then aggregated these local scores into a global consensus motif using F-MoDA (Yaish and Orenstein 2025). The resulting motif displayed a clear preference for the sequence “UGCAUG,” which aligns well with the established consensus reported in the CISBP-RNA database (Fig. 6A). The recovered motif achieved a MoSBAT similarity score of 0.757.

We next applied the same analysis to the *Mus musculus* ETS-family DBP Gm5454 (UP00423). Using the top 100 DNA sequences (Fig. 12B, available as supplementary data at *Bioinformatics* online), we recovered the canonical ETS binding core. This result is consistent with the known binding preference reported in the UniPROBE database (Fig. 6B), with a MoSBAT similarity score of 0.719, further demonstrating that NucProNet accurately captures the intrinsic sequence determinants required for DNA binding.

Over all NBPs in the dataset, NPBIP achieved a mean MoSBAT similarity score of 0.212±0.266 for RBPs and 0.388±0.278 for DBPs (Fig. 13, available as supplementary data at *Bioinformatics* online). Individual similarity scores for each NBP, alongside visual comparisons of recovered versus ground-truth logos over all NBPs in the dataset, are available in the GitHub repository.

## 4 Discussion

In this work, we developed NPBIP, the first method designed to directly solve the *NewProNewSeq* problem: predicting the binding affinity of uncharacterized NBPs to any given nucleic-acid sequence. NPBIP integrates two complementary components: SimBind, which leverages sequence similarity to known NBPs, and NucProNet, a deep-learning model that learns protein–nucleic-acid interactions directly from sequence. By combining these two components, NPBIP achieves accurate and generalizable predictions for both RBPs and DBPs, outperforming *k*-mer-based methods and its components. Beyond predictive performance, NPBIP can uncover nucleic-acid binding preferences in a visual format. Using occlusion analysis, we successfully recovered canonical motifs for both RBPs and DBPs, confirming that NucProNet learns accurate intrinsic binding preferences.

To contextualize NPBIP’s performance (mean Pearson correlation of r=0.414 over RNAcompete and 0.581 over PBM), we compared it to settings where experimental data for the test NBP are available and to the correlation between experimental replicates as an empirical upper bound. In the *NewSeq* task, where the test NBP’s experimental data is available, state-of-the-art methods, such as MultiRBP and our MultiDBP, achieve mean correlations of 0.686±0.17 and 0.727±0.16, respectively. Our analysis of replicate experiments, comprising 64 pairs for RNAcompete and 10 for PBM, revealed mean correlations of 0.542±0.29 and 0.818±0.10, respectively. NPBIP approaches these experimental ceilings without any prior information regarding the query NBP.

One of the key conclusions from this work is that *k*-mer models, though useful summaries of binding preferences, fail to capture all the information that determines binding. This limitation is clearly evident in methods like JPLE; while its approach effectively transfers binding preferences into *k*-mer profiles, the subsequent transition from these intermediate models to scoring experimental probes appears to introduce a performance bottleneck. NPBIP achieves superior performance since it learns the complex patterns and rules of NBP–nucleic-acid binding directly from the full sequence. Another finding of this work is the improvement gained by aggregating over methods and over NBPs. First, the integration of SimBind and NucProNet was significantly better than either component alone. Second, this same principle held true within SimBind, where aggregating information from the top *m* similar NBPs improved prediction performance compared to using only the single most similar NBP. We conclude that a single method or a single similar NBP can be a noisy predictor, but the collective signal from multiple sources provides a much more robust and accurate result.

We identified two primary limitations in NPBIP. First, the performance of both SimBind and NucProNet is strongly correlated with the query NBP’s maximum similarity to the training set (Fig. 5B). This suggests that the deep-learning model also implicitly relies on similarity and, consequently, that NPBIP’s predictions will be less reliable for NBPs from novel families that are highly dissimilar from any training NBP. Our leave-family-out analysis further supports this, highlighting generalization to novel families remains an open challenge. Second, NPBIP is based on models that were trained on RNAcompete and PBM data, which raises the risk of over-fitting to the specific artifacts and biases of these experimental assays. As a result, the prediction performance of NPBIP may, in part, reflect its ability to learn and model data-specific characteristics rather than generalizable predictive power.

Several future directions are promising. First, NPBIP currently relies exclusively on microarray-based binding data. However, other high-throughput *in vitro* experimental assays, such as HTR-SELEX and HT-SELEX, offer complementary datasets that could enrich training and improve generalization across experimental assays. Second, integrating structural information, such as protein structures predicted by AlphaFold (Jumper *et al.* 2021) and RNA secondary structure predictors, could allow NPBIP to better reflect the biophysical nature of NBP–nucleic-acid binding, improving both prediction performance and robustness. Third, extending our interpretability framework to the protein sequence to identify individual amino acids involved in binding remains a significant direction for future research.

## Supplementary Material

btag232_Supplementary_Data

## Data Availability

The datasets analyzed in this study are publicly available. Raw PBM data can be accessed via the UniPROBE database (https://thebrain.bwh.harvard.edu/uniprobe/). RNAcompete datasets are available through the Hughes Lab website (https://hugheslab.ccbr.utoronto.ca/supplementary-data/RNAcompete_eukarya/ and https://hugheslab.ccbr.utoronto.ca/supplementary-data/RBPZoo/). The source code and processed datasets are hosted on GitHub at https://github.com/OrensteinLab/NPBIP.
