# Supplementary material for: NPBIP: predicting binding preferences of uncharacterized nucleic-acid-binding proteins
Source: Bioinformatics. 2026 Jul 7;42(Suppl 1):btag232. doi: 10.1093/bioinformatics/btag232 (PMC13341135; doi:10.1093/bioinformatics/btag232)
Supplement: btag232_Supplementary_Data [file btag232_supplementary_data.pdf]

Supplementary Information for:

NPBIP: Predicting binding preferences of uncharacterized nucleic-acid-binding proteins

Noam Shimshoviz[1], Safwan Butto[2] and Yaron Orenstein[1,2,*]

[1]The Mina and Everard Goodman Faculty of Life Sciences, Bar-Ilan University, Ramat Gan, Israel
[2]Department of Computer Science, Bar-Ilan University, Ramat Gan, Israel

[*]Corresponding author: yaron.orenstein@biu.ac.il

Supplementary Methods

NucProNet

Protein tower

The protein encoder processes the amino-acid sequence using frozen per-residue embeddings from the ESM-2 model (hidden size $D_{\mathrm{ESM}} = 5120$). Given a sequence of length L_p, the embeddings are mean-pooled to obtain a global representation $\mathbf{h}_p \in \mathbb{R}^{D_{\mathrm{ESM}}}$. This vector is then projected through a two-layer multilayer perceptron (MLP):

$$\mathbf{z}_p = \mathrm{MLP}(\mathbf{h}_p) = f_2(\mathrm{LayerNorm}(\mathrm{GELU}(f_1(\mathbf{h}_p)))) ,$$

where f_1 and f_2 are linear layers and GELU is the activation function. The final output is layer-normalized to produce the protein embedding $\mathbf{e}_p \in \mathbb{R}^D$.

Nucleic-acid tower

Each nucleotide of an L_r-long nucleic-acid sequence is mapped to a token embedding vector $\mathbf{t}_r \in \mathbb{R}^D$ and combined with learned positional encodings:

$$\mathbf{X}_0 = \mathrm{Embed}(\mathrm{tokens}) + \mathrm{PosEnc}(L_r).$$

A cascade of three dilated convolutional blocks with kernel sizes of 5, 9, and 13 (inspired by DLPRB's kernel length of 5 and increasing by 4 in subsequent layers) and dilation rates to extract local motif-like features:

$$\mathbf{X}_i = \mathrm{ConvBlock}_i(\mathbf{X}_{i-1}), \quad i = 1, 2, 3.$$

Each block applies a 1D-convolution with the same number of input and output channels (D), and padding chosen such that the output preserves the input length L_r. Each block includes GELU activation, LayerNorm, dropout, and a residual connection, ensuring that both the dimensionality and sequence length remain unchanged.

An additional 9-kernel convolutional layer is applied and added to the residual stream with a learnable scalar weight γ. This size was fixed to capture typical 6–10 nt biological motifs (and fits the k-mer size of 9 and 10 in RNAcompete and PBM probe design, respectively):

$$\mathbf{X}_{\mathrm{res}} = \mathbf{X}_3 + \gamma \cdot \mathrm{Conv1d}_{k=9}(\mathbf{X}_3).$$

A two-layer transformer encoder then models global dependencies:

$$\mathbf{X}_{\mathrm{tf}} = \mathrm{TransformerEncoder}(\mathbf{X}_{\mathrm{res}}).$$

Finally, a GatedPooling layer computes a weighted average over sequence positions:

$$\mathbf{h}_r = \sum_{l=1}^{L_r} \alpha_l \, \mathbf{X}_{\mathrm{tf},l},$$

where the attention weights α_l depend on both sequence features and a learnable Gaussian positional bias:

$$\alpha_l = \mathrm{softmax}\left(\mathbf{w}^\top \mathbf{X}_{\mathrm{tf},l} - \frac{\alpha(l-c)^2}{2\sigma^2}\right),$$

with learnable parameters α and σ, and $c = (L_r - 1)/2$ denoting the sequence midpoint. The final output is layer-normalized to produce the nucleic-acid embedding $\mathbf{e}_r \in \mathbb{R}^D$.

Cosine bilinear head

Given the protein embedding $\mathbf{e}_p \in \mathbb{R}^D$ and nucleic-acid embedding $\mathbf{e}_r \in \mathbb{R}^D$, the Gated Bilinear Low-Rank Cosine Head computes their interaction score as:

$$s_{pr} = \mathbf{u}_p^\top \mathbf{v}_r + b,$$

where

$$\mathbf{u}_p = \mathrm{norm}\Big(U\mathbf{e}_p \odot \big(1 + \lambda \, \tanh(G\mathbf{e}_p)\big)\Big), \quad \mathbf{v}_r = \mathrm{norm}(V\mathbf{e}_r).$$

Here, $U, V, G \in \mathbb{R}^{D \times R}$ are learnable projection matrices, λ is a gating strength scalar, and $\mathrm{norm}(\cdot)$ ensures cosine similarity between the projected vectors. The low-rank structure (rank $R = 512$) enables efficient modeling of cross-modal interactions between protein and nucleic-acid features.

The final output is a matrix $\mathbf{S} \in \mathbb{R}^{B_p \times B_r}$, where each entry corresponds to the predicted binding affinity between a protein–nucleic-acid pair.

Computing infrastructure and implementation

We implemented NPBIP in PyTorch ($v2.0+$) and trained it on NVIDIA A100 (80 GB VRAM) GPUs. We performed hyper-parameter optimization via Optuna, with individual training trials typically concluding within $60 - -75$ minutes. To run NPBIP locally, users require approximately 85 GB of disk space to store the ESM-2 (15B) model weights. Due to the scale of ESM-2, a peak memory footprint of 85 GB (RAM) is required during the initial loading phase. Once the model is initialized, inference for a single NBP against $10,000$ sequences takes approximately 25 seconds on the aforementioned hardware.

Comparison of k-mer score aggregation strategies

To evaluate the impact of the k-mer aggregation strategy, we compared four strategies: mean, sum, max, and a biophysically-inspired Boltzmann-sum aggregation (partition function approach):

$$Score = \ln\left(\sum_{i=1}^{L-k+1} e^{\beta \cdot Z_i}\right) \tag{1}$$

where L is the probe length, k is the k-mer length, and β is an inverse-temperature scaling parameter optimized on a validation set. To determine the optimal value of β, we performed a line search on the validation set independently for each k-mer model. Specifically, we evaluated a predefined set of candidate values, $\beta \in \{0.1, 0.5, 1, 5, 10, 20, 30, 50, 60, 70, 80, 90, 100\}$, and selected the value that maximized the Pearson correlation between predicted and observed binding intensities. For each test RBP, we evaluated all four aggregation strategies across all k-mer-based models. The mean strategy achieved the highest performance, with an average Pearson correlation of 0.339 ± 0.084 (averaging the four k-mer-based methods). The mean strategy significantly outperformed the Boltzmann-sum approach in three out of the four methods ($p < 0.001$, Wilcoxon signed-rank test; Supplementary Figure S12). Thus, we selected the simple mean aggregation strategy for all models in this study.

Supplementary Tables

**Category**	**Hyper-parameter**	**NucProNet**	**NucProNet-k-mer**	**Search range**
Towers Output Dimension	D	512	256	{64, 128, 256, 384, 512}
Protein tower	MLP hidden layer size	256	256	{128, 256, 512}
	Dropout	0.3889	0.3558	[0.1, 0.4]
Nucleic-acid tower	Conv block 1 (kernel, dilation)	(5, 1)	(5, 1)	Fixed
	Conv block 2 (kernel, dilation)	(9, 2)	(9, 2)	Fixed
	Conv block 3 (kernel, dilation)	(13, 4)	(13, 4)	Fixed
	Conv block 4 (kernel)	(9)	(9)	Fixed
	Transformer layers	2	1	[1, 4] (Integer)
	Transformer heads	4	4	Fixed
	Dropout	0.2653	0.2540	[0.1, 0.4]
Scoring head	Head rank ($RANK$)	256	768	{128, 256, 512, 768}
Training	Loss function correlation weight	0.6522	0.7996	[0.5, 0.9]
	Optimizer	AdamW	AdamW	Fixed
	Learning rate (LR)	0.000267	7.61e-05	$[10^{-5}, 10^{-3}]$ (Log)
	Global weight decay	0.000386	0.0352	$[10^{-4}, 10^{-1}]$ (Log)
	Protein MLP weight decay	0.000125	3.43e-05	$[10^{-5}, 10^{-3}]$ (Log)
	Protein batch size	8	8	Fixed
	Nucleic-acid batch size	2048	2048	Fixed
	Training steps	10,709	13,275	[1000, 15000] (Integer)

Table S1. Final hyper-parameters for the NucProNet and NucProNet-k-mer models, optimized on the RNAcompete validation set. The search range indicates the values or distributions sampled during optimization.

Supplementary Figures

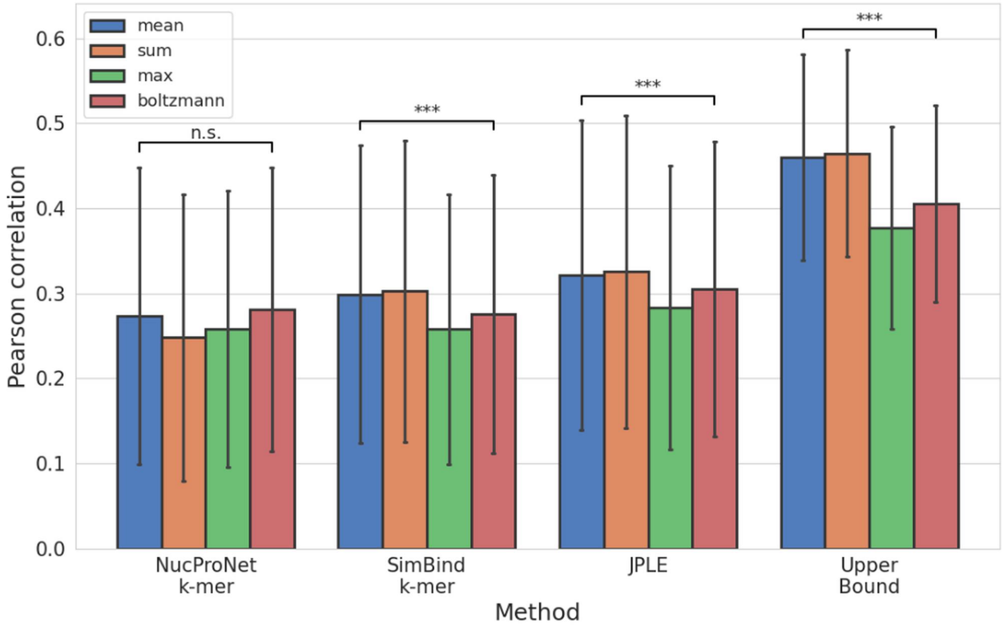

Supplementary Figure S1: Comparison of aggregation strategies for converting k-mer-level scores to probe-level intensities for RNA-binding proteins. We evaluated four strategies for each k-mer-based method: mean score (mean), sum of scores (sum), maximum score (max) and Boltzmann-sum (boltzmann). Error bars represent standard deviation. Statistical significance between the mean and Boltzmann-sum was assessed using a Wilcoxon signed-rank test (*** denotes $p < 0.001$)

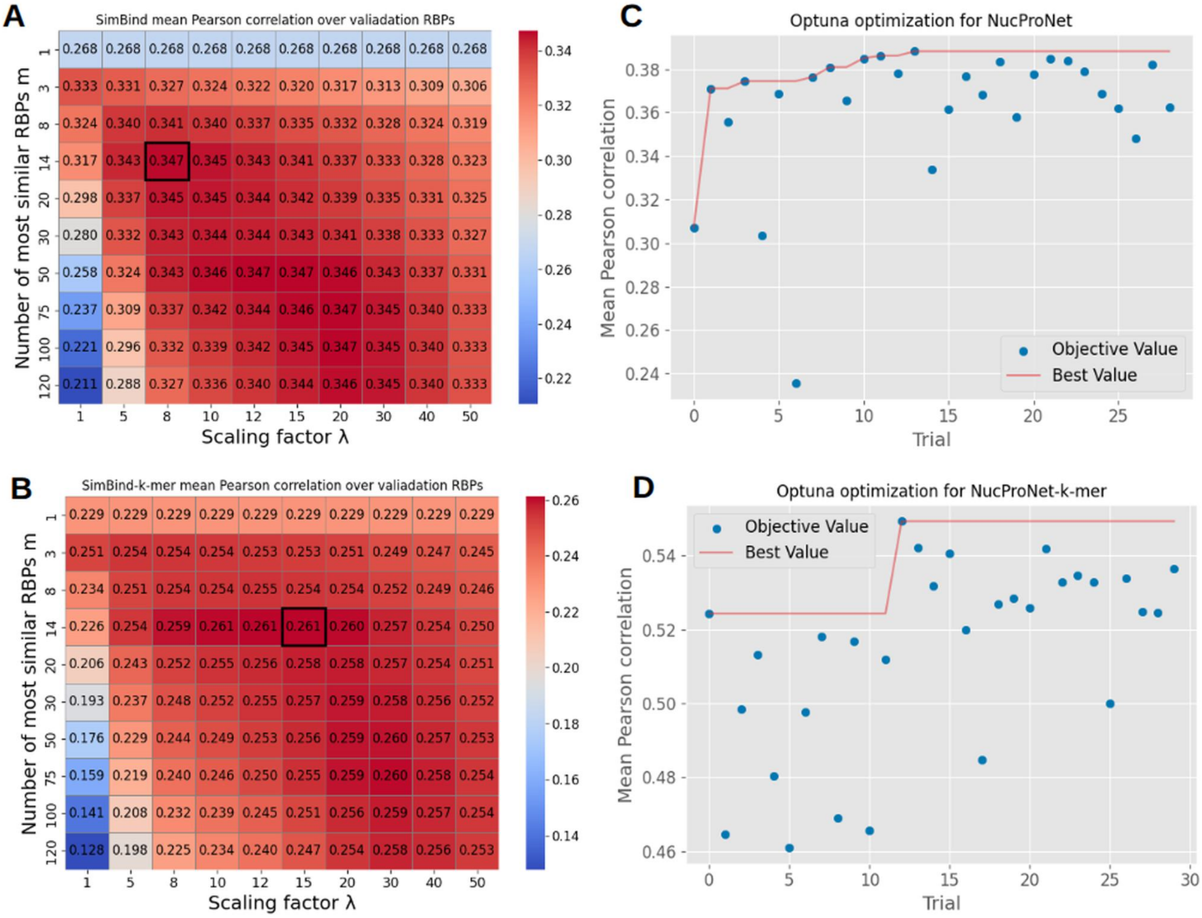

Supplementary Figure S2: Hyper-parameter search on the RNAcompete validation set. (A) Grid search for SimBind hyper-parameters. (B) Grid search for SimBind-k-mer hyper-parameters. (C) Optuna search results for NucProNet. (D) Optuna search results for NucProNet-k-mer.

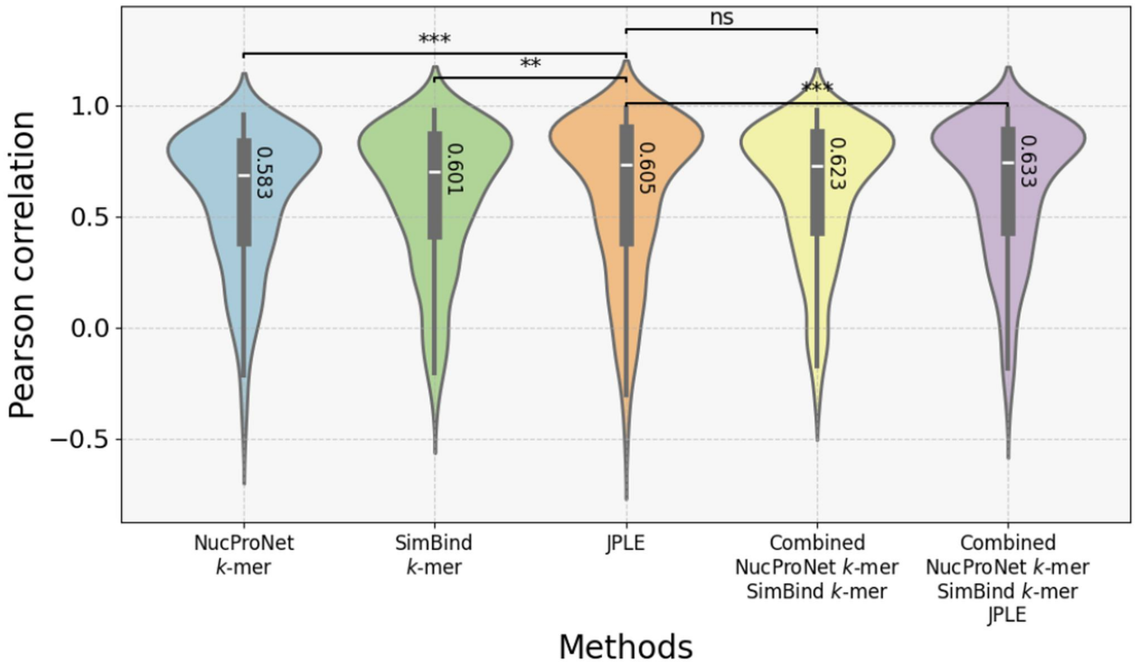

Supplementary Figure S3: Performance in predicting RBP 7-mer models. Distribution of Pearson correlations on the experimental 7-mer models for RBPs. Mean correlations are written. (*** denotes $p < 0.001$, ** denotes $p < 0.01$, Wilcoxon signed-rank test).

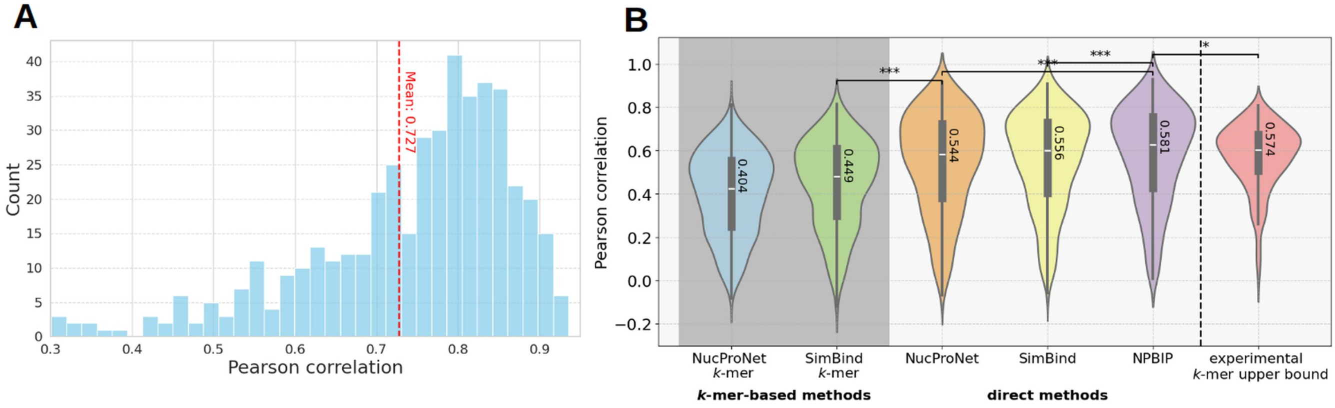

Supplementary Figure S4: Performance in predicting DNA binding. (A) Distribution of Pearson correlation achieved by MultiDBP. (B) Distribution of Pearson correlations on the test proteins. Mean correlations are written. (*** denotes $p < 0.001$, * denotes $p < 0.05$, Wilcoxon signed-rank test).

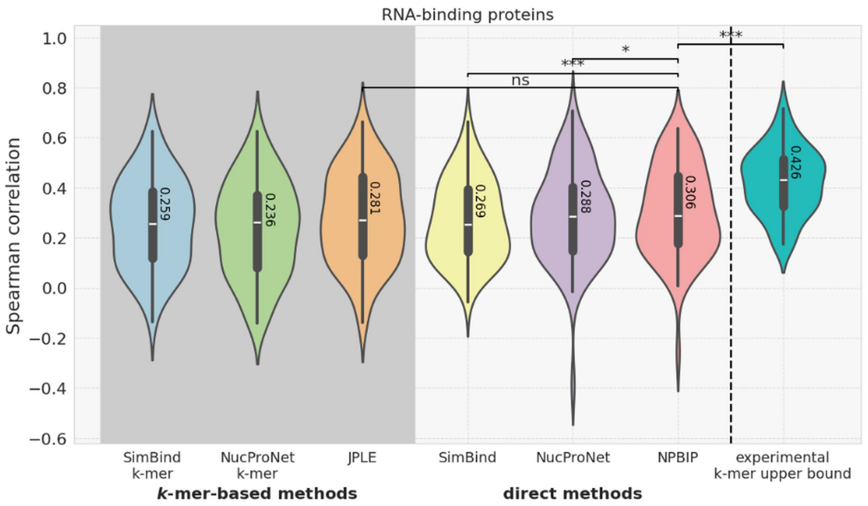

Supplementary Figure S5: Prediction performance in predicting RNA binding. Distribution of Spearman correlations over the test RBPs. Mean correlations are written. (*** denotes $p < 0.001$, ** denotes $p < 0.01$, * denotes $p < 0.05$, Wilcoxon signed-rank test).

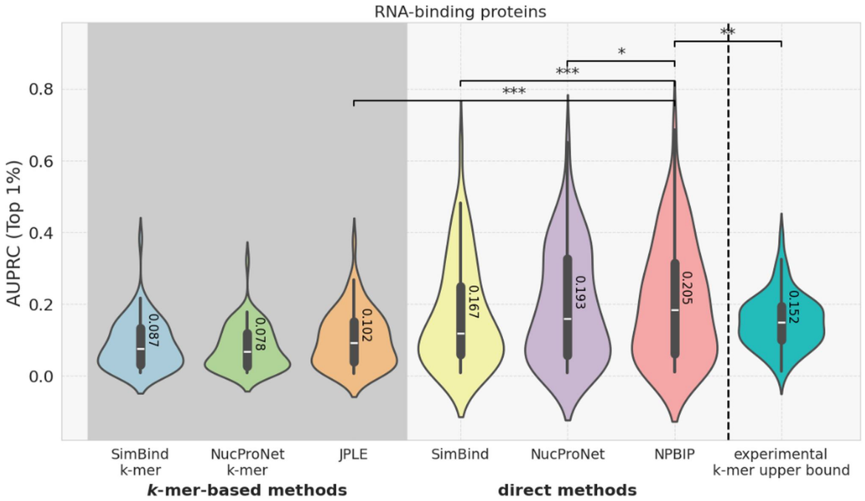

Supplementary Figure S6: Prediction performance in predicting RNA binding. Distribution of AUPRC (Top 1%) over the test RBPs. AUPRC (Top 1%) represents the Area Under the Precision-Recall Curve, calculated by treating the top 1% of sequences with the highest observed binding intensities as the positive class. Mean values are written. (*** denotes $p < 0.001$, ** denotes $p < 0.01$, * denotes $p < 0.05$, Wilcoxon signed-rank test).

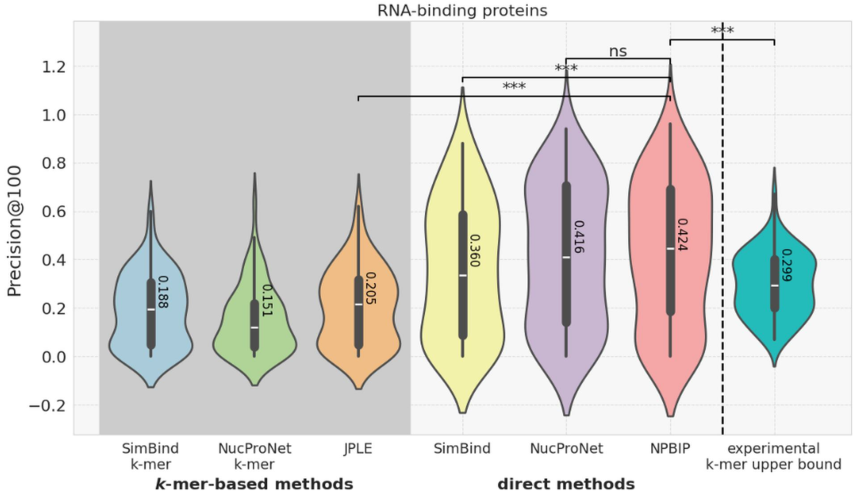

Supplementary Figure S7: Prediction performance in predicting RNA binding. Distribution of Precision@100 over the test RBPs. Precision@100 represents the fraction of the 100 highest-predicted sequences that are correctly identified among the top 100 experimental ground-truth sequences. Mean values are written. (*** denotes $p < 0.001$, ** denotes $p < 0.01$, * denotes $p < 0.05$, Wilcoxon signed-rank test).

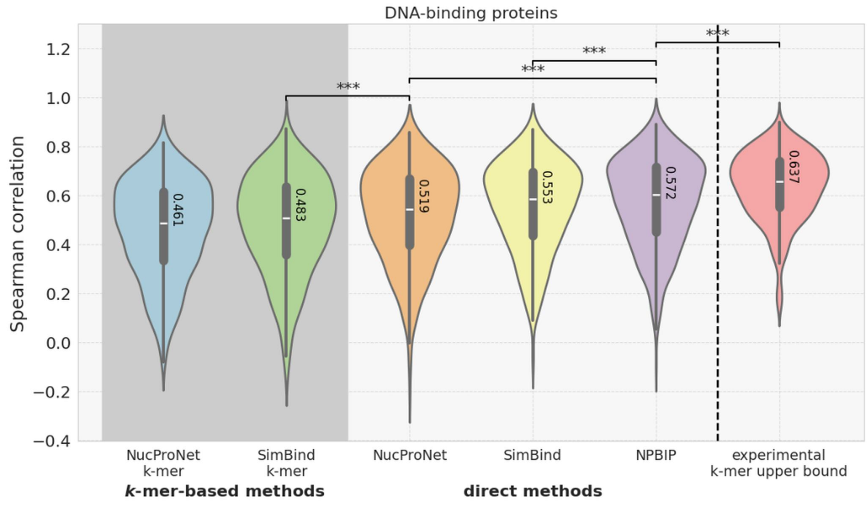

Supplementary Figure S8: Prediction performance in predicting DNA binding. Distribution of Spearman correlations over the test DBPs. Mean correlations are written. (*** denotes $p < 0.001$, ** denotes $p < 0.01$, * denotes $p < 0.05$, Wilcoxon signed-rank test).

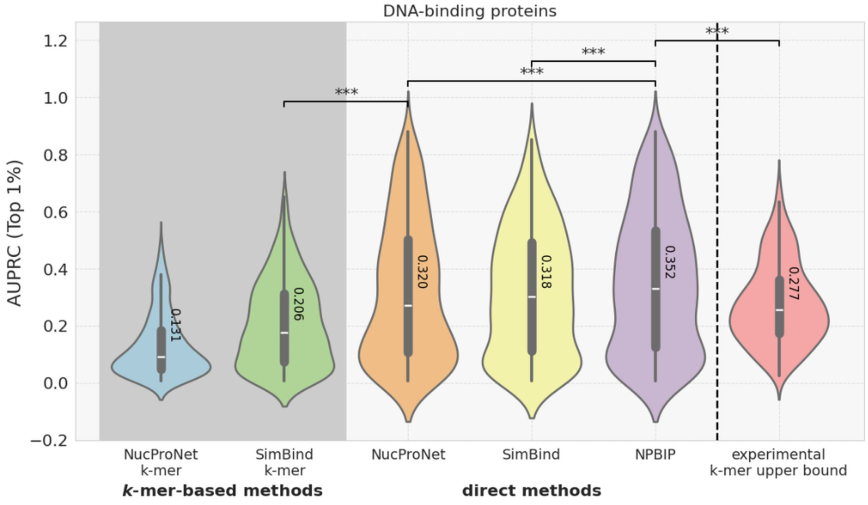

Supplementary Figure S9: Prediction performance in predicting DNA binding. Distribution of AUPRC (Top 1%) over the test DBPs. AUPRC (Top 1%) represents the Area Under the Precision-Recall Curve, calculated by treating the top 1% of sequences with the highest observed binding intensities as the positive class. Mean values are written. (*** denotes $p < 0.001$, ** denotes $p < 0.01$, * denotes $p < 0.05$, Wilcoxon signed-rank test).

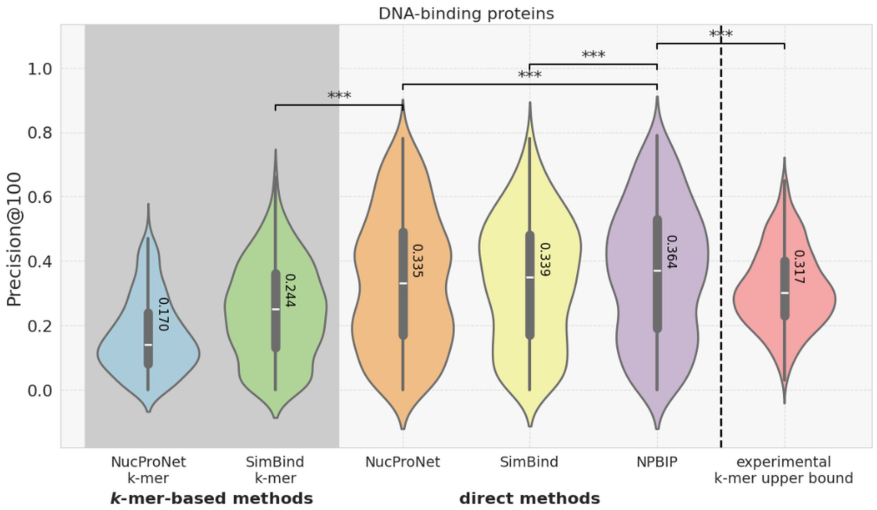

Supplementary Figure S10: Prediction performance in predicting DNA binding. Distribution of Precision@100 over the test DBPs. Precision@100 represents the fraction of the 100 highest-predicted sequences that are correctly identified among the top 100 experimental ground-truth sequences. Mean values are written. (*** denotes $p < 0.001$, ** denotes $p < 0.01$, * denotes $p < 0.05$, Wilcoxon signed-rank test).

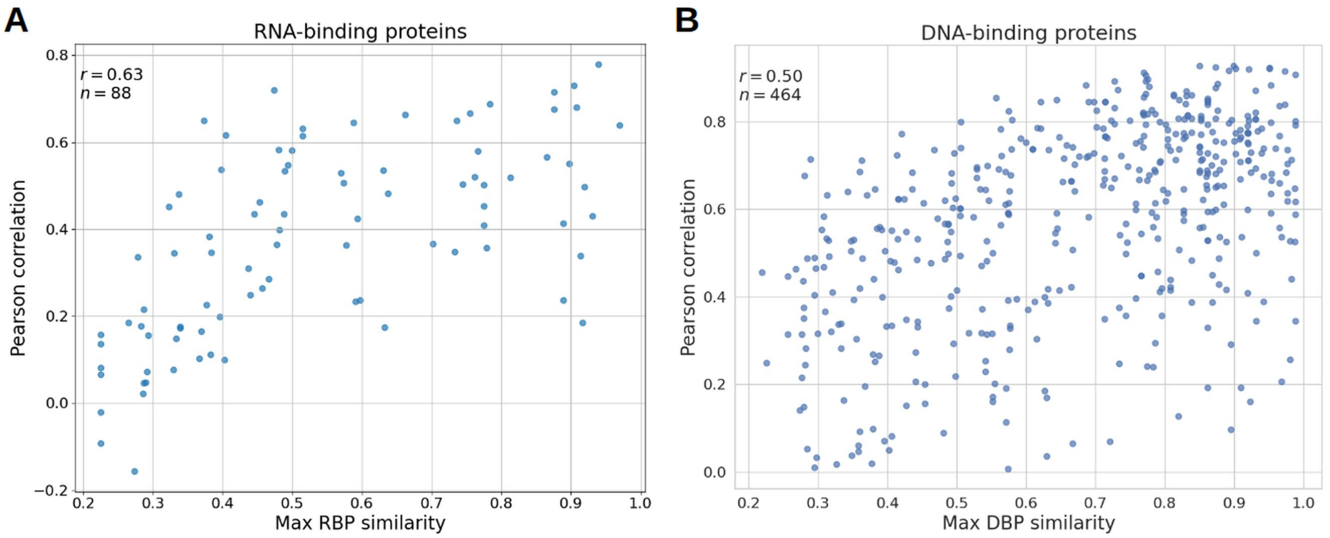

Supplementary Figure S11: NPBIP performance as a function of maximum query–training NBPs similarity (A) over 88 RBP test experiments, and (B) over 464 DBP experiments.

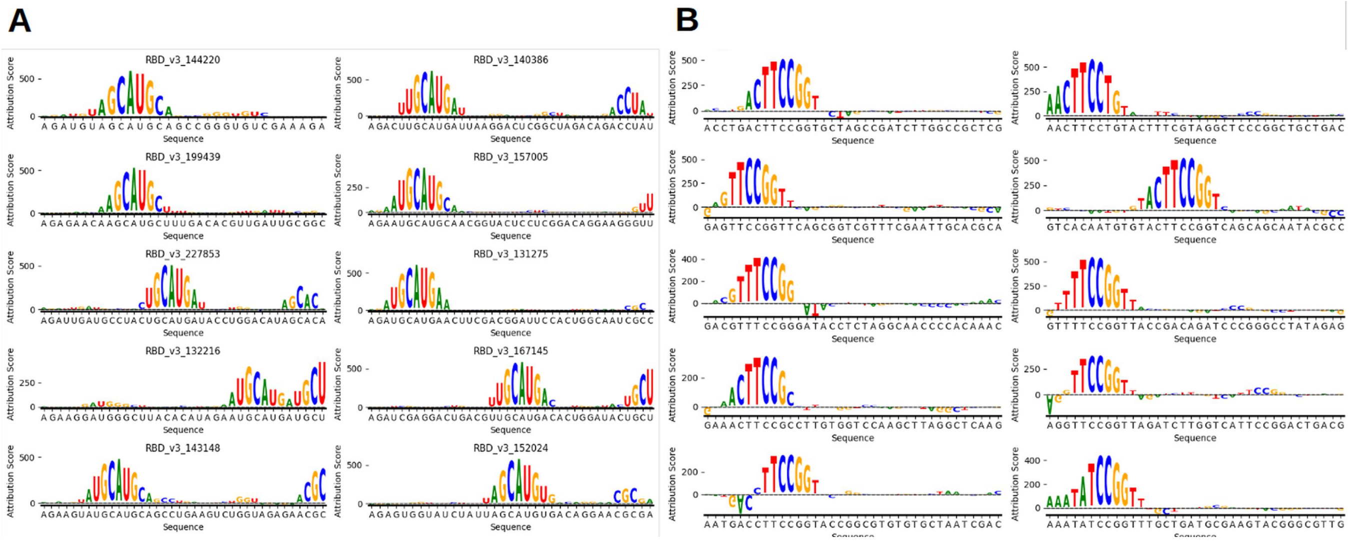

Supplementary Figure S12: Visualization of NucProNet attribution scores for 10 top-predicted sequences (A) for RBFOX2, and (B) for Gm5454.

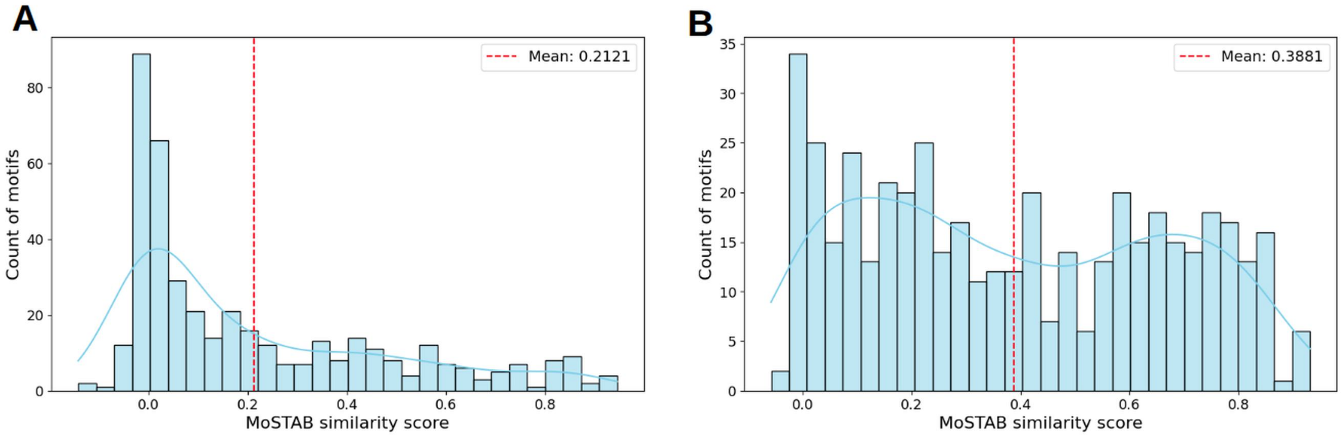

Supplementary Figure S13: Distribution of MoSBAT similarity scores between motifs predicted by NucProNet (via F-MoDA) and ground-truth motifs (A) 420 RBP experiments. (B) 464 DBP experiments.
